# Different influences of phylogenetically conserved and independent floral traits on plant functional specialization and pollination network structure

**DOI:** 10.3389/fpls.2023.1084995

**Published:** 2023-01-24

**Authors:** Ganju Xiang, Yunyi Jiang, Jinmao Lan, Liuying Huang, Lijun Hao, Zhiqian Liu, Jing Xia

**Affiliations:** ^1^ Hubei Provincial Key Laboratory for Protection and Application of Special Plant Germplasm in Wuling Area of China, College of Life Sciences, South-Central Minzu University, Wuhan, Hubei, China; ^2^ State Key Laboratory of Biocatalysis and Enzyme Engineering, Hubei Collaborative Innovation Center for Green Transformation of Bio-Resources, Hubei Key Laboratory of Industrial Biotechnology, School of Life Sciences, Hubei University, Wuhan, China

**Keywords:** phylogenetically conserved floral traits, phylogenetically independent floral traits, floral size, flower density, plant specialization, pollination network

## Abstract

Plant specialization and pollination network structure play important roles in community assembly. Floral traits can mediate plant–pollinator interactions and thus have important impacts on nestedness and modularity of pollination network. When such traits are phylogenetically conserved, therefore, phylogeny and traits should predict network structure to similar degrees. Moreover, conserved network structures were also found attributed to pollination syndrome or pollination system. However, we still know little about the relation between pollination syndrome and pollination network, especially under a phylogenetic framework. Herein, we established a phylogenetic framework including five floral traits (flower density, floral size, floral shape, floral symmetry, and floral color) and five species-level metrics (species strength, weighted closeness, specialization *d*’, nestedness contribution, and modularity contribution) to test how floral traits could directly or indirectly influence species’ specialization and network structure in central China. Phylogenetic signals were found in all floral traits except flower density. Structural equation model and phylogenetic structural equation model results showed that both floral size and floral density affected plant specialization and its contribution to network modularity indirectly. However, compared with phylogenetic independent flower density, phylogenetic conserved floral size had much more complexed influences, having a direct influence both on species’ specialization and on modularity contribution. In this nested and modular network, abundant species with larger flowers tend to be more central and had larger values of *z*. Floral shape, symmetry, and color could act as co-flowering filters in pollination sharing and help to shape network modularity. Our results emphasize that phylogenetically conserved traits partially represent pollination syndrome and are important drivers for modular structure of local pollination network. This study may improve the understanding how the evolutionary history and ecological process drive local network structure and dynamics.

## Introduction

About 90% of angiosperm species depend on animal pollinators for reproduction (Ollerton et al., 2011). As the well-known mutually beneficial relationship, plant–pollinator interactions are paramount in angiosperm diversity and the maintenance of ecosystem services (Stebbins, 1970; Bascompte and Jordano, 2007; Gallai et al., 2009; Doré et al., 2021; Wei et al., 2021). More importantly, the worldwide threats to biodiversity may, in turn, affect plant–pollinator interactions (González-Varo et al., 2013). Therefore, it is an everlasting topic to understand the patterns and causes of plant–pollinator networks and their dynamics, especially in conservation and restoration (Lara-Romero et al., 2019). Intensive studies have demonstrated that both ecological variables and functional traits as well as their past evolutionary history may explain such network patterns (Bascompte and Jordano, 2007; Petanidou et al., 2008).

Floral traits are one of the most important determinants of network patterns by mediating interactions with floral visitors (Junker et al., 2013; Watts et al., 2016; Lara-Romero et al., 2019; Lázaro et al., 2020; Suárez-Mariño et al., 2022). First, phenotypic complementarity or morphological match may determine to what degree plants and pollinators can interact with each other in the community and, thus, the realized connectance and species’ position in pollination network (Rezende et al., 2007a; Stang et al., 2007). For example, local flower abundance is important for the realization of pairwise interactions (Carstensen et al., 2014). More abundant plants and those with larger flowers showed higher linkage levels and important roles in the plant–pollinator network in a rich coastal community (Lázaro et al., 2020). Plants with radially symmetrical flowers also had more within-module connections than species with bilaterally symmetrical flowers in networks cross Western Canada (Chamberlain et al., 2014). Second, trait matching between partners in mutualistic interactions also influences species’ specialization, which plays important roles in species assembly, evolution, and stability of biological communities (Tinoco et al., 2017; Zhao et al., 2019; Lara-Romero et al., 2019; Albor et al., 2022; Pardo-De la Hoz et al., 2022). For instance, flowers pollinated by humming birds in southeastern Peru had the longest corollas and the highest level of complementary specialization and exclusivity in plant–pollinator networks (Watts et al., 2016). Villalobos et al. (2019) also found that morphological mismatching due to floral symmetry generated high levels of reciprocal specialization in plant–pollinator networks at Glenbow Ranch Provincial Park. Finally, as flowers are complex structures, each of these distinct trait classes (abundance, phenology, signals/cues, morphology, and resources) can mediate interactions with floral visitors. It thus demands a more holistic view to assess the effect of floral traits on network metrics. For example, Suárez-Mariño et al. (2022) revealed floral similarity had a significant interaction effect on pollinator sharing with flowering overlap and an indirect influence of on network nestedness as a consequence. However, we cannot tell which traits are more important than others using the floral similarity method. Hence, causality analysis [e.g., structural equation model (SEM)] among multiple traits data and network metrics at the species level will help to explain which and how floral traits directly or indirectly influence species’ specialization and thus their network contributions in a plant–pollinator network.

Furthermore, evolutionary processes are also revealed as important drivers of local network structure and dynamics (Guimarães et al., 2017; Segar et al., 2020). Similarity among species in traits related to ecological interactions is frequently associated with common ancestry (Aizen et al., 2016; Guimarães et al., 2017; Segar et al., 2020). Plants sharing common ancestry (similar traits) thus tend to interact with largely overlapping ecological assemblages of pollinators, and *vice versa* (Gómez and Perfectti, 2010; Cirtwill et al., 2020). When traits involved in mediating species interactions are phylogenetically conserved, therefore, phylogeny and traits should predict network structure to similar degrees (Gómez and Perfectti, 2010; Kantsa et al., 2018; Villalobos et al., 2019). At network level, some studies revealed more importance of pollinator traits and phylogenetic history in determining network structure (Rezende et al., 2007b; Reverté et al., 2016), whereas others found the reverse (Chamberlain et al., 2014). At the species level, however, only few networks showed important roles of phylogeny for traits in determining species-level network metrics (Chamberlain et al., 2014). In addition, rare studies have been conducted to explain this pattern under a phylogenetic framework, although measuring of phylogenetic signal can provide a good means to quantify the influence of phylogeny.

Many studies focused on pollination network from the view of plant rather than pollinators. Actually, selection of pollinator functional groups should lead to convergence of floral traits among unrelated plant species, which is known as pollination syndrome or pollination system (Willmer, 2011; Carstensen et al., 2016; Reverté et al., 2016; Dellinger, 2020). Furthermore, spatial rewiring of interactions could be constrained by pollination systems, resulting in conserved network structures despite high variation in pairwise interactions (Carstensen et al., 2016). However, we still know little about the relation between pollination syndrome and connectivity, nestedness, or modularity of pollination network. For instance, to what extent floral traits are likely related to pollination syndrome? How such traits contribute to specialization and/or network modularity in different plant communities?

Herein, we collected plant–pollinator network data over 3 years in a community in central China. Then, we established a phylogenetic framework including five floral traits (flower density, floral size, floral shape, floral symmetry, and floral color) and five species-level metrics (species strength, weighted closeness, specialization *d*’, nestedness contribution, and modularity contribution) to test whether floral traits could directly or indirectly influence species’ specialization and network structure. Specially, we addressed the following questions: (a) Are these floral traits related to pollinator foraging preferences? (b) Whether phylogenetic constraints had effects on floral traits and species-level network metrics? (c) How floral traits could directly or indirectly influence species’ specialization and network contributions in a plant–pollinator network under a phylogenetic framework. This study will help to highlight the role of floral traits in shaping the pollination network structure, especially when the floral traits are related to pollination syndrome.

## Materials and methods

### Study site

This study site was located in the Qizimeishan National Nature Reserve (30.041565°N, 109.77305° E, 1,800–1,900 m; **Supplementary Figure 1**), Hubei Province, China, which is rich in biodiversity in monsoon-dominated continental East Asia (Man et al., 2008; Li et al., 2021). Hubei Qizimeishan National Nature Reserve is a forest ecosystem nature reserve and mainly protects mid-subtropical forest ecosystem and rare and endangered wild fauna and flora, which is on the IUCN Green List (https://iucngreenlist.org/explore/green-list-sites/). The annual average temperature and precipitation in the study site is 8.9°C and 1876.0 mm, respectively (Man et al., 2008). In July and August, the forest margin herb community is composed of many co-flowering herbaceous and shrub species, which provide an ideal system to conduct studies on plant–pollinator interactions. We collected data on plant–pollinator interactions, combining plots and transect sampling method (Chamberlain et al., 2012). Along a track from the Nature Reserve to the town Chunmuying, field works were repeated in six transects, which were separated from each other by more than 0.5 km. To characterize plant–flower visitor interactions in an unbiased manner, we established 50 permanent plots (3 × 3 m for grassland plot or 5 × 2 m for roadside plot) in 2017 for a long-term pollination observation at the community level. An alternative plot was re-established near the origin one when it was destroyed or when the distribution ranges changed among years. As a result, pollination observation was conducted in a total 9–12 plots for each transect in each year, which could include all the flowering species to the greatest extent possible. The total area observed in each year was 510 m^2^ in 2017, 537 m^2^ in 2018, and 458 m^2^ in 2019, respectively. Finally, 66 insect-pollinated plant species from 28 families were identified and included in our pollination network (**Supplementary Table 1**).

### Field observations of flower pollinators

Field observations were carried out during July and August 2017, 2018, and 2019. To build the pollination networks, we conducted all surveys at 3-day intervals for each transect. Pollination observations were arranged in 15-min observation periods from 0900 h to 1700 h in sunny and cloudy days when pollinators were active. Three plots were surveyed simultaneously with two or three observers each plot, recording the number of pollinators foraging each plant species during an observation period for one plot. In addition to the relatively abundant species, we also took care to sample the rare co-flowering species in the plot, ensuring to record as possible more plant–pollinator interactions as we could (Albor et al., 2022). A visitor that encountered the anther or stigma of a flower was considered a pollinator. Morphological species were recorded in the field, and specimens were identified to the lowest taxonomical level possible by specialists later. Voucher specimens of insects and plants were kept in South-Central Minzu University, Wuhan, China. To reduce the sampling bias due to differences in foraging-time among pollinators, plots in each transect were observed as a given sequence every day, and, finally, observations for each plot included morning, midday, and afternoon-periods. We focused on the diurnal pollination network and thus did not record nocturnal pollinators in the field work because rare species were pollinated at night in this study. In total, 1,255 hours of observations were assigned in 3 years (152.5 hours on 12–21 August 2017; 571.25 hours on 10 July to 21 August 21 2018; and 531.25 hours on 7 July 7 to 25 August 25 2019).

### Measurement of floral traits

We collected the following traits for plants species: flower density, floral size, floral shape, floral symmetry, and floral color. Flower density was defined as the floral abundance of a species divided by the area of plots (**Supplementary Table 1**). We used flower density instead of flower abundance because it played a more important role in pollinator attraction especially when mass-flowering plants were distributed mostly patchily. Here, we counted the numbers of open attractive units (flowers or inflorescences) per plot during field pollination observation (see **Supplementary Table 1** for details of attractive unit for each species; Lázaro et al., 2020). For Asteraceae species, a capitula is considered as an attractive unit except for *Artemisia lavandulifolia* and *Eupatorium lindleyanum*, in which a spike and a synflorescence (small capitula numerous in apical dense corymb) as the unit respectively (**Supplementary Table 1**).

For 61 focal species, we conducted size measurement on at least 30 fully opened flowers/inflorescence (the same attractive floral units as that used to estimate flower density) from 10 randomly selected individuals in 2021 and 2022. For each species, we measured width, height, and tube length (if possible) of a flower unit, using a digital vernier caliper. As the simplest measure that can be compared among all species, the width/diameter of a flower/inflorescence was finally used to assess the effect of floral size on network metrics at the species level (**Supplementary Table 1**; Lázaro et al., 2020). For *Houttuynia cordata*, the diameter of involucral bracts was measured instead of the width of inflorescences. For the other five species (*A. lavandulifolia*, spike width; *Astilbe chinensis*, panicle width; *Cryptotaenia japonica*, umbellule diameter; *Heracleum hemsleyanum*, umbellule diameter; *Hydrangea strigose*, cyme width), in which inflorescences act as the attractive units, inflorescence size was collected from Flora of China (http://www.iplant.cn/foc) because only the flower size was measured in the field.

Floral shape was categorized into four types as follows: open dish/bowl, open tube, flag or gullet, and tube (**Supplementary Table 1**). 1) Open dish/bowl: Species have exposed nectar and pollen, or pollen presented as pollinia. This group included species in Adoxaceae, Apiaceae, Apocynaceae, Araliaceae, Caprifoliaceae, Celastraceae, Gentianaceae, Geraniaceae, Hydrangeaceae, Hypericaceae, Epilobium, Primulaceae, Ranunculaceae, and Rosaceae. 2) Open tube: Species have a head of small ray and disc tubular flowers such as Asteraceae species or with densely flowered capitula or spike such as *Dipsacus asper* and *Polygonum* species. Stamens and pistils are exposed, and nectar are concealed at the base of narrow tubes. 3) Flag or gullet: Species have exposed stamens and pistils but concealed nectar at the bottom of narrow or wide tubes, including Fabaceae, Gentianaceae, Lamiaceae, Ranunculaceae (*Aconitum hemsleyanum*), Commelinaceae, Boraginaceae, and Primulaceae. 4) Tube: Species have deep narrow or wide corolla tube, hidden pollen, and concealed nectar such as in Campanulaceae, Scrophulariaceae, Gentianaceae, Asparagaceae, Balsaminaceae, Liliaceae, and Cucurbitaceae. In general, both pollen and nectar are easy access for open dish/bowl and open tube flowers. However, flag-, gullet-, and tube-shaped flowers are always mechanically strong.

Flower symmetry was categorized as zygomorphy or actinomorphy for each species. Asteraceae species have actinomorphic inflorescences (attractive unit) with zygomorphic flowers and thus were assigned as actinomorphy. Furthermore, we recorded floral color of each species in the field, which was recognized as five types: white/pink, yellow, yellow green, blue, or purple.

### Network metrics at the species level

To obtain a global overview of plant–pollinator network structure, a quantitative visitation network was constructed from the interaction data pooled together sites and years, because insect abundance, diversity, and the plants that they use may vary among years (Ouvrard et al., 2018). Rather than on species identity, networks based on insect functional groups can reveal patterns in the functionality and sustainability of complex plant–pollinator communities when studied across gradients or replicates (Fontaine et al., 2006; Geslin et al., 2013; Koski et al., 2015). Therefore, we categorized insect visitors into 12 functional groups according to their body size and foraging behavior. That is: six Hymenoptera types (ants, ANT; bumblebees, BB; large solitary bees, LL; honeybees, HB; small bees, SB; wasps, WASP), two Diptera types (hoverflies, HF; other flies, FL), two Lepidoptera types (butterflies and moths except hawkmoths, BF; hawkmoths, HM), one Coleoptera type (beetles, BT), and other visitors (see **Supplementary Table 3** for details of pollinator groups).The strength of each interaction was identified by the number of flower pollinators in a particular functional group that were observed visiting a focal plant species (**Supplementary Figure 2**).

The following five network metrics at the species level were calculated from the plant–pollinator network. (1) Species strength is the sum of dependencies of each species, with higher value indicating more pollinator functional groups depending on it (see the works of Watts et al., 2016; Suárez-Mariño et al., 2022). (2) Weighted closeness centrality is calculated as the sum of the number of shortest distances between the species in question and all other species in the network, with all ties weighted as 1/(link weight/average link weight in the network) (Opsahl et al., 2010). Low closeness scores indicate specialization, and high closeness scores indicate nodes (pollinators) are more “central”, e.g., closer to all other species in the network. (3) Specialization *d*’ calculates how strongly a species deviates from a random sampling of interacting partners available, based on the observed interaction frequencies (Blüthgen et al., 2006). It ranges between 0 for extreme generalization and 1 for extreme specialization, respectively (Olesen et al., 2007). (4) Nestedness contribution estimates the individual contribution of each plant to the overall nested structure of the network (Saavedra et al., 2011; Suárez-Mariño et al., 2022). (5) Modularity contribution, namely, *z* value in the network, evaluates the individual contribution from each plant species to entire network modularity (Guimeraà et al., 2005; Suárez-Mariño et al., 2022). The first three metrics quantify plant specialization, and the last two metrics refer to its consequences on network structure. All network metrics were calculated by the “bipartite” package in *R*.

In addition, *z* values (within-module degree) and *c* values (among-module degree) were also calculated. Moreover, linear models (LMs) detected no significant relationship between *c* values and *z* values in the plant–pollinator network (*t* = 0.601, *P* = 0.55). Thus, *z* value for each species can be considered as its modularity contribution in the network (Suárez-Mariño et al., 2022). Similar with the previous studies, weighted versions of *z* and *c* were calculated using species strength instead of species degree here (Watts et al., 2016). To objectively define thresholds, 100 null models for original networks were run quantiles 95 (*q95*) as critical *c* and *z* values were employed. At the same time, we also computed quantiles 50 (*q50*) of the *c* and *z*, respectively. The *c* and *z* values were all calculated using *cz*values function of *R* based on the quantitative modularity (*Q*), which was estimated by the QuanBiMo to algorithm (Dormann et al., 2009; Dormann and Strauss, 2014).

For each plant species, a topological role in the network was then assigned on the basis of the shape of the *c* and *z* frequency distribution in the network (Lázaro et al., 2020). A network hub (*z_i_* ≥ *z*_q95_, *c_i_* > *c*_q50_) is highly linked to species within their own module and species of other modules, which is important for the connectivity among species both within its own module and within the network (Olesen et al., 2007). Whereas, a module hub (*z_i_* ≥ *z*_q95_, *c_i_* ≤ *c*_q50_) plays an important role in connecting species within its own module. A connector species (*z_i_* < *z*_q95_, *c_i_* > *c*_q50_) is crucial for among-module connectivity but plays an inferior role within its own module. Peripheral species (*z_i_* < *z*_q95_, *c_i_* ≤ *c*_q50_) have a few interactions inside their own module and rarely link to any other modules.

### Statistical analysis

#### Whether phylogeny effect on floral traits and plant–pollinator networks

To test the influence of phylogeny on floral traits and network metrics at the species level described above, a phylogenetic tree was first constructed on the basis of mega-phylogeny of plants by the packages “V.PhyloMaker” in *R* (Qian and Jin, 2016; **Supplementary Figure 3**). Then, phylogenetic signals were calculated using Blomberg’s *K* for all quantitative parameters (flower density, floral size, and each of the network metrics at the species level) and using Pagel’s *λ* for the discrete trait (floral shape, symmetry, and color). Floral shape was coded as follows: 1 for open dish/bowl; 2 for open tube; 3 for flag or gullet; 4 for tube, with larger value more difficult to access for pollinators with short mouthparts (Geslin et al., 2013). Floral symmetry was coded as follows: 1 for actinomorphy and 2 for zygomorphy. Similarly, floral color was valued from light color to dark as follows: 1 to white/pink, 2 to yellow, 3 to yellow green, 4 to blue, and 5 to purple, respectively. *K* measures the extent to which a trait displays phylogenetic signal using the variance of the standardized phylogenetically independent contrasts as a measure of how well the tree fits the data given a Brownian motion (BM) model of trait evolution (Blomberg et al., 2003). It indicates no phylogenetic signal, where *K* = 0, suggests the trait distribution perfectly conforms to BM, where *K *= 1, and indicates stronger similarities among closely related species than expected under BM, where *K* > 1. Pagel’s *λ* coefficient reflects the phylogenetic dependence of observed trait data with respect to a pure Brownian model of evolution (Pagel, 1999), with the value varying from 0 (no phylogenetic signal, phylogenetically independent) to 1 (phylogenetically conserved, the distribution of trait values across the phylogeny is exactly as expected under BM). *K* was computed with the function *phylosignal* in package “picante” (Kembel et al., 2010) and *λ* with the function *fitDiscrete* in package “geiger” (Harmon et al., 2008).

#### How flower traits influence plant–pollinator networks

In this study, five species-level network metrics were divided into two groups: plant specialization (species strength, weighted closeness, and specialization *d*’; see Tinoco et al., 2017; Suárez-Mariño et al., 2022) and their contributions in the network structure (nestedness contribution and modularity contribution; see Suárez-Mariño et al., 2022). First, we calculated a covariance matrix of five floral traits and five network metrics by the *R* package “corrplot” (Wei and Simko, 2017) to provide an estimate of the correlation between the floral traits with network metrics. Only flower density and floral size had significant relationships with network metrics (**Supplementary Figure 4**). To assess the direct and indirect effects of floral traits on plant–pollinator network structure, we then conducted a piecewise SEM for phylogenetically independent ecological floral traits (flower density) and a phylogenetic SEM (PSEM) for phylogenetically conserved floral traits (floral size, shape, symmetry, and color), respectively. By joining multiple variables into a single causal network, SEM is a useful tool for quantifying both direct and indirect effects (Lefcheck, 2016).

SEMs comprised generalized LMs (GLMs; by the *stat* function in *R* package “nlme”) with normal distribution and identity link (Shipley, 2009, Shipley, 2013), whereas the PSEMs comprised phylogenetic generalized least squares (PGLS; using the *gls* function in the *R* package “nlme”) to account for evolutionary dependence among species (Garland et al., 1993; Martins and Hansen, 1997; Wei et al., 2021). In each model, floral traits were thought as predictor variables to be directly or indirectly related to the network metrics. Because plant specialization could directly influence the network structure (Suárez-Mariño et al., 2022), we also used plant specialization metrics as direct predictor variables for metrics of the network structure in the two models. Moreover, we could not presume the relationships among network metrics to be causal in each group, and they were defined as being correlated errors (Lefcheck, 2016).

The goodness-of-fit of each model was evaluated using two-sided Fisher’s *C* statistic based on Shipley’s d-separation (directed separation) test of conditional independencies (Shipley, 2009, Shipley, 2013). Because PGLS generalizes the independent contrasts approach and can be used to incorporate a variety of models of evolutionary change (Garland et al., 1993; Martins and Hansen, 1997), the *model.sel* function in the *R* package “MuMIn” (Barton, 2018) was used to select the best one from the following evolutionary models: (1) BM, which traits evolve according to random drift; (2) Pagel’s lambda (PL), which the rate of trait evolution is optimized from the data; and (3) Ornstein–Uhlenbeck (OU), which traits evolve toward an optimum. PSEMs were then conducted with the best evolutionary model (**Supplementary Table 2**).

In this study, all data were summarized as the means ± standard errors, and all statistical tools were run in *R* with version 4.2.0 (R Core Team, 2022). The significance was considered to occur at a level of 0.05.

## Results

**Figure 1:**
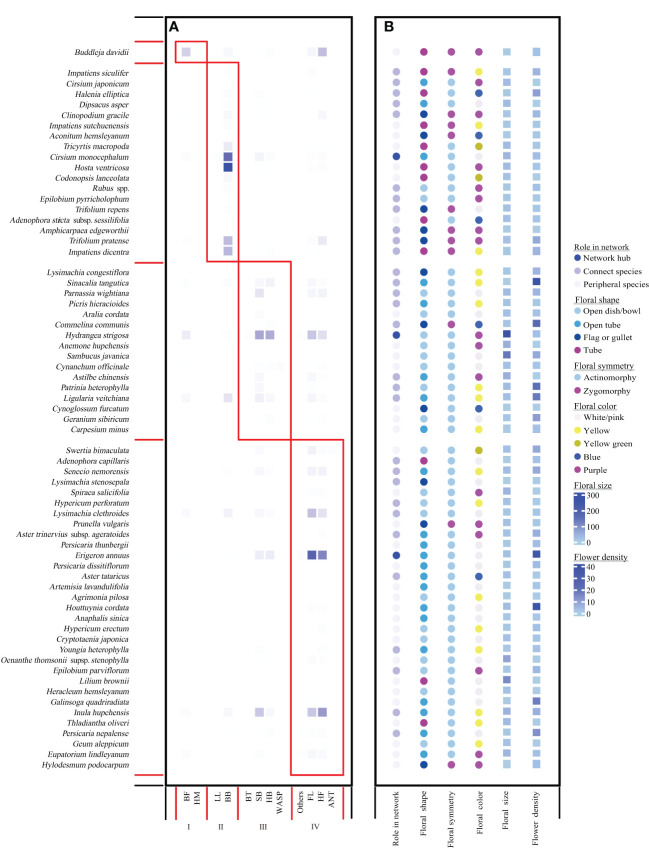
Network modular structure **(A)** with plant species’ roles in the network and floral traits as shown in **(B)**. **(A)** Four modules delineated by red boxes, which was calculated using *computeModules* functions in the *R* package “bipartite”. The color intensity indicates the interaction frequency between partners. Species are sorted according to their modular affinity; plants as rows and pollinators as columns. **(B)** Role in network of each species, and their flower density, floral size, floral shape were shown as different color. The heatmap of flower density and floral size was drawn using the package “complexHeatmap” (Gu et al., 2016) in *R*.

In our 3-year field surveys, we tracked a total of 62,683 pollinator individuals. Among the most abundant pollinator groups were bees (bumblebees, 30.897%; small bees, 13.367%; honeybees, 7.55%), flies (hoverflies, 20.778%; other flies, 21.406%), and lepidopterans (butterflies and moths except hawkmoths, 4.580%). The plant–pollinator network exhibited a significant nested structure [weighted nestedness metric based on overlap and decreasing fill (WNODF) = 64.466] (Almeida-Neto et al., 2008) and also a significant modular structure (*Q* = 0.405) with four modules identified (**Figure 1**). Lepidoptera species (BF and HW) were classified into module I, whereas bumblebees and large solitary bees such as carpenter bees were classified into module II. Honeybees and small bees such as Halictidae and Andrenidae, as well as beetles and wasps, were grouped into module III, whereas Diptera species (HF and FL) and other tiny insects (ANT and others) were classified into module IV.

Only *Buddleja davidii* was included in module I. In module II, bumblebees and carpenter bees preferred to forage on flag-, gullet-, or tube-shaped flowers that are more mechanical, bilateral flowers, and bule to purple flowers (**Figure 1**; **Supplementary Figure 5**). Whereas, plants grouped into module III were those with significant larger flowers and greater flower density (**Supplementary Figure 5**). Furthermore, plant species in module IV always had flowers with light color such as white/light pink and yellow (**Figure 1**, **Supplementary Figure 5**). Across the 66 plant species, 33 peripheral species and 30 connector species were identified, respectively (**Figure 1**). However, only three network hubs were detected and assigned to *Cirsium monocephalum* in module II, *Hydrangea strigose* in module III, and *Erigeron annuusm* in module IV, respectively (**Figure 1**). Moreover, network hubs had significantly larger floral size than connector and peripheral species (**Figure 1**, **Supplementary Figure 6**).

A significant phylogenetic signal was found in floral size (Bloomberg’s *K* = 0.6138, *P* = 0.009), floral shape (Pagel’s *λ* = 1), floral symmetry (Pagel’s *λ* = 1), and floral color (Pagel’s *λ* = 0.8162), based on the consensus tree for the 66 plant species in the network (**Supplementary Figure 3**). In contrast, there were no significant phylogenetic signals in flower density (flower density: Bloomberg’s *K* = 0.1488, *P* = 0.496) and neither in network metrics (species strength: Bloomberg’s *K* = 0.0777, *P* = 0.874; weighted closeness: Bloomberg’s *K* = 0.0860, *P* = 0.837; specialization *d*’: Bloomberg’s *K* = 0.1594, *P* = 0.457; nestedness contribution: Bloomberg’s *K* = 0.2135, *P* = 0.107; modularity contribution: Bloomberg’s *K* = 0.0604, *P* = 0.958; respectively).

**Figure 2:**
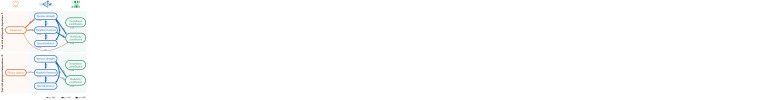
The SEMs showing how floral traits influence network metrics in the transect. **(A)** shows that the pattern of floral size, with phylogenetic constraints, has influences on the plant–pollinator network by PGLS, and **(B)** shows that flower density, with phylogenetic independence, has effects on the plant–pollinator network *via* GLMs. Paths among variables included in the model are shown. The solid arrows indicate a positive effect of a variable on another. Standardized path coefficient was given on each arrow.

Both PSEM and SEM adequately represented the data and support the hierarchical structure proposed in the model (PSEM: Fisher’s *C* = 1.433, *P* = 0.488, Akaike information criterion (AIC) = 49.433; SEM: Fisher’s *C* = 3.714, *P* = 0.446, AIC = 29.714). Floral size, the phylogenetically conserved floral trait, showed significant positive effects on species strength and weighted closeness (**Figure 2A**). In contrast, only weighted closeness was significantly positively influenced by flower density, the phylogenetically independent floral trait (**Figure 2B**). Plants with larger flowers showed higher species strength, meaning that more pollinator functional groups depend on it. Species with larger flowers and higher flower density were associated with higher closeness centrality in the network, indicating decreased plant specialization. Unexpectedly, however, higher specialization *d*’ was also related to larger floral size and flower density indirectly, because network metrics of species’ specialization had significant positive relationships with each other (**Figure 2**).

Species strength and weighted closeness, rather than *d*’, had significant positive effects on modularity contribution in both models (**Figure 2**). The results suggested floral size and flower density could indirectly affect the topological role of a plant species in the network *via* its direct effects on species strength and closeness centrality. In addition, our result also revealed a direct and positive influence of floral size on modularity contribution in the PSEM (**Figure 2A**). In contrast, neither floral traits nor plant specialization was found to be correlated with nestedness contribution (**Figure 2**). No significant relationship between nestedness contribution and modularity contribution was detected, either. This result indicated that the floral traits listed here did not contribute to the nested structure of plant–pollinator network in the study community.

## Discussion

### Pollination system

Pollinators use floral traits such as size, shape, symmetry, and color to locate and discriminate between different co-flowering species in the community (Chittka and Raine, 2006). Therefore, different functional pollinators may have innate preferences for certain shapes and colors (Bascompte et al., 2003; Fontaine et al., 2006; Geslin et al., 2013; Reverté et al., 2016). In line with other studies, we did find similar pollinator foraging preferences in the study community (**Figure 1**; **Supplementary 5**). For example, long-mouthpart pollinators such as lepidoptera and bumblebees preferentially foraged on more mechanical flowers with tubular corollas in module I and module II, whereas short-mouthpart ones such as honeybees, solitary bees, and flies tend to forage on flowers with open-corolla and radial symmetry (Wignall et al., 2006; Geslin et al., 2013). Furthermore, our study also revealed color preferences of functional pollinators: bumblebees favor blue and purple, whereas flies favor white and yellow (**Figure 1**). Moreover, honeybees and small bees also preferred the most mass-flowering ones with significant largest flowers (**Figure 1**, **Supplementary Figure 5**). To some extent, this finding is consistent with what pollination syndrome theory and pollination system described (Faegri and Van der Pijl, 1979; Carstensen et al., 2016; Dellinger at al., 2019). However, our results also indicate that pollinators made their foraging decisions due to mixed traits rather than a single trait, because plant species with a single similar trait did not attract similar pollinator assemblages (Reverté et al., 2016).

As expected, flower density reflects local resource abundance, which is determined by neutral ecological process rather than phylogeny history (Violle et al., 2007). He et al. (2019) defined density as an ecosystem trait to link functional traits such as other floral traits to macroecology. In contrast, floral morphological traits are often phylogenetically conserved but also community dependent (Blomberg et al., 2003; Chamberlain et al., 2014; Reginato and Michelangeli, 2016). For example, Chamberlain et al. (2014) found that flower symmetry (radial and bilateral) was most frequently phylogenetically conserved, whereas flower size was less (phylogenetic signal detected in 41% and 28% of the total trees, respectively). In most cases, however, previous studies indicated that phylogenetic signal for floral color was lacking (except in the works of Shrestha et al., 2014; Reverté et al., 2016). In our community, all of the floral morphological traits measured (size, shape, symmetry and color) showed to be phylogenetical dependent (**Supplementary Figure 3**), meaning that related species had similar floral morph in the focal community.

Although the floral shape, symmetry, and color played important roles in flower choices of functional pollinators, they did not affect either network metrics measured (**Supplementary Figure 4**). Beyond that, both phylogenetically conserved floral size and phylogenetically independent flower density had significant influences on plant specialization and thus plant–pollinator network structure (**Figure 2**). However, some differences were also detected between PSEM and SEM. To some extent, therefore, the results implied different impacts of phylogenetically conserved and independent floral traits on plant functional specialization and thus its consequences on network structure. Furthermore, unexpected positive relations were revealed between complementary specialization *d*’ and the other two indices measured as species strength and weighted closeness, which means a species may both be central in the network (a generalist) but also be a specialist ranked by *d*’. The implications of these results will be discussed in detail in the next sections.

### Different influences of phylogenetically independent and conserved floral traits on plant functional specialization

In addition to the regular pollinators, more rewarding plants (e.g., more flower abundance and high local flower density) may increase occasional visitors, enhancing pollinator sharing with less abundant plants (Makino et al., 2007; Carvalheiro et al., 2014). As a result, these species tended to have a greater influence on the pollination of co-flowering plant species, resulting in more central and decreased functional specialization (Lázaro et al., 2020). In consistent with the hypothesis, the SEM confirmed that phylogenetic independent flower density was directly positively related with weighted closeness in our network (**Figure 2B**). The PSEM revealed that the floral size had positive influences on both species strength and weighted closeness (**Figure 2A**). Similar relationships were found in that of Lázaro et al. (2020), which suggested a direct positive related with the linkage level of plant species and an indirect positive relation with closeness centrality in a diverse dune marshland. However, it is important to note that our result indicated the effect of floral size after controlling for the effects of phylogenetic relatedness by PGLS. This means that both phylogeny and floral size determined species’ position in the network. Closely related plant species had similar floral size, and those with larger size can attract more pollinator functional groups and also occupy central positions in the plant–pollinator network. This result is expected because floral displays that vary in shape, size, color, height, and scent can act as attraction signals for flower visitors (Campbell et al., 2012; Gibson et al., 2012; Junker et al., 2013, Carvalheiro et al., 2014). Furthermore, we did not find out any correlations between other phylogenetic conserved floral traits (shape, symmetry, and color) and plant functional specialization and its consequences on network structure (**Supplementary Figure 4**). However, these morphological traits may be related to pollinator preference and thus lead to the modularity structure which will be discussed in the later section.

Finally, different metrics were used in the network analysis that might be correlated among themselves (Watts et al., 2016; Lázaro et al., 2020). A species with higher complementary specialization *d*’ means that it has more specialized plant–pollinator interactions and low pollinator sharing in a plant–pollinator network, and *vice versa*. Therefore, there is always a negative correlation between complementary specialization *d*’ and other specialization metrics such as species degree, species strength, and closeness centrality (Lázaro et al., 2020; Suárez-Mariño et al., 2022). However, some studies also revealed an opposite pattern, an unexpected positive relationship between complementary specialization *d*’ and weighted closeness (Pocock et al., 2011; Trøjelsgaard et al., 2019; Gaiarsa and Bascompte, 2022). It seems like a “paradox” because low closeness scores indicate specialization and high closeness scores more central (e.g., closer to all other species in the network). Whereas, our results also revealed such a paradox (**Figure 2**). Species with larger floral size and flower density were quantified as moderately specialized by complementary specialization *d*’, although they were the most centralized participants in the networks and were considered as high generalization when quantifying specialization with the other two indices.

In our study system, species with larger flower and patchily mass-flowering (high flower density) occupied central positions in the network, especially the three network hubs (*C. monocephalum* in module II, *H. strigose* in module III, and *E. annuusm* in module III), which had significantly larger floral size (**Supplementary Figure 5**). Larger flowers can enhance floral attraction to pollinator visitation (Willmer, 2011), and thus, all functional pollinator groups were found foraging on them. However, most interactions actually occurred only with one or several focal functional groups (**Supplementary Figure 2**). As a result, the complementary specialization *d*’ ranked them as high specialism, which refer exclusively to the interaction frequencies relative to the availability of the partners and completely ignore the actual number of partners (Blüthgen et al., 2006; Watts et al., 2016; Pardo-De la Hoz et al., 2022). As a contrast, rare species with smaller flowers were more likely “opportunists” and visited by the most common flower visitor(s) in the focal community (Koski et al., 2015). In line with other studies, therefore, our study also convinces that specialization indices convey different concepts of specialization and hence quantify different aspects (Watts et al., 2016). Regardless, just as Watts et al. (2016) suggested, it requires careful consideration when defining a specialist.

### Different influences of phylogenetically independent and conserved floral traits on network structure

Plants offering more resources are likely to be visited by more pollinators and thus more likely to influence another indirectly *via* shared pollinators that, in turn, may result in plant–pollinator network modular structure (Kunin, 1997; Cartar, 2009; Bartomeus et al., 2013; Carvalheiro et al., 2014). Several studies have convinced that floral abundance had direct or indirect effects on species’ closeness in their study communities (Sazima et al., 2010; Watts et al., 2016; Lázaro et al., 2020) and that species strength showed a strong direct or indirect association with the modular structure of plant–pollinator networks (Watts et al., 2016; Suárez-Mariño et al., 2022). Similarly, our results also revealed flower density can indirectly positively influence the modularity contribution of each plant species *via* its direct positive effects on species strength and/or weighted closeness (**Figure 2**). Floral size was important in the three network metrics at the species level measured except the nestedness contribution. Not only the indirect influences but also a direct effect of floral size on modularity contribution was detected in the PSME model (**Figure 2**). Floral size was significant positively correlated with *z* value (**Figure 2**). In our community, species with larger flowers had larger values of *z* or interacted more within their modules. In contrast, a reverse relationship was found across Canadian plant–pollinator communities (Chamberlain et al., 2014). This inconsistency suggests inconstant effects of floral size of a particular species on its contribution to network modularity, which may be pollination network context dependent.

In a pollination network context, pollination syndromes and their corresponding functional group of pollinators could contribute to modularity structure because interactions within pollination systems principally occur inside modules (Carstensen et al., 2016). For example, co-flowering species can filter pollinators *via* floral traits such as size, shape, symmetry, and color, because they may act as barriers to certain pollinators and thus drive pollinator preferences (Stang et al., 2006; Stang et al., 2009; Wei et al., 2021). Our results did reveal that different functional groups had different foraging preferences. For the six most frequent pollinators, butterflies and bumblebees with longer mouthparts mostly prefer to visit flag, gullet, or tubular bule/purple flowers that are more mechanical but did not distinguish floral symmetry, whereas others (small bees, honeybees, hoverflies, and other flies) showed preferences on white and yellow flowers that are radial and open access for pollinators (**Figure 1**, **Supplementary Figure 5**). Moreover, honeybees and small bees were also mostly frequently found to visit on larger flowers with high local density than the others (**Supplementary Figure 5**). Therefore, module organization in our network is partly caused by convergent trait sets including floral shape, symmetry, color, and size. Such a co-evolutionary unit may describe the relationship between interacting species and give insights into the dynamics of ecological communities (Olesen et al., 2007).

For the whole network in a diverse community, it represents the community-wide interactions where niche partitioning in pollinator use and asymmetric facilitation may confer fitness advantage of rarer species (Wei et al., 2021). For the sub-network within module, however, it more likely describes how common and rare species (low local flower density) may interact with each other when pollinator niches overlap. Then, facilitative interactions among plants *via* pollinator sharing may favor rare plant species (Moeller, 2004; Wei et al., 2021). Furthermore, previous studies revealed that only occasional visitors might increase with local flower density (Makino et al., 2007). Rare species may thus benefit from both pollinator attraction and traits if it was growing with abundant hub species grouped into different modules. For example, rare species with smaller attractive unit were valued significantly lower specialization *d*’ in the focal community, partly indicating that they were more likely to be foraged by the most common pollinators attracted by common species (Koski et al., 2015). Hence, sub-networks may help us to understand the mechanisms behind which evolutionary and ecological factors determined plant–pollinator interactions and their changes across space and time (Carstensen et al., 2016).

Both nestedness and modularity are thought to provide benefits for ecological communities (Fortuna et al., 2010). Inconsistent with network modularity, nestedness contribution was determined neither by floral traits nor species-level specialization metrics (**Figure 2**), although the study network exhibited both a significant nested structure and a significant modular structure. No significant relationship between nestedness contribution and modularity contribution was detected, either. This finding indicated other unmeasured traits such as phenological factors, to some degree, accounting for the nested structure of plant–pollinator network in the study community. For instance, Suárez-Mariño et al. (2022) showed that an increase in flowering overlap led to a higher degree of plant generalization and, in turn, with consequences for network nestedness. Furthermore, pollinators may play a more active role in the definition of interaction identity than plants because of pollinator mobility (Bascompte and Jordano, 2007). As a result, related animal species are more likely to share host plants than related plant species are to share pollinator visitors (Reverté et al., 2016). In agreement with the findings of Rezende et al. (2007b), our results suggested pollinators might be of even greater importance than plants on nestedness structure in our network. For example, more generalized pollinator species were found likely responsible for higher nestedness (Chesshire et al., 2021).

## Conclusions

Both floral size and floral density showed direct and indirect effects on plant specialization and its contribution to network modularity in a diverse community in Central China. However, compared with phylogenetic independent flower density, phylogenetic conserved floral size had much more complexed influences, having a direct influence both on species’ specialization and on modularity contribution. In this nested and modular network, abundant species with larger flowers tend to be more central and had larger values of *z*. Floral shape, symmetry, and color, as well as the other phylogenetic conserved traits, could act as co-flowering filters in pollination sharing and help to shape the modular structure. Our results emphasize that phylogenetically conserved traits partially represent pollination syndrome and are important drivers for modular structure of local pollination network. This study may improve the understanding how the evolutionary history and ecological process drive local network structure and dynamics.

## Data availability statement

The original contributions presented in the study are included in the article/**Supplementary Material**. Further inquiries can be directed to the corresponding author.

## Author contributions

JX conceived and designed the experiments. JX, YJ, LYH, LJH, and ZL performed the experiments. GX analyzed the results. YJ made and revised charts. JX and GX wrote the first draft of the manuscript. All authors contributed to the revision and experimental design of the study. All authors read and approved the submitted version.

## Supplementary material

Supplementary Figure 1 Study sites, grassland plots **(A, B)** and roadside plots **(C, D, E, F)** in Hubei Qizimeishan National Nature Reserve, China.

Supplementary Figure 2 The plant-pollinator network in our community with pollinator at the top and plants at the bottom. The widths of the lines connecting plants with their pollinators represents the number of flowers visited by each pollinator. Plant species were shown as plant code (See **Supplementary Table 1** for each species’ code). Pollinator and plant species were sequenced according to the module structure.

Supplementary Figure 3 The phylogenetic tree of 66 plants included in the network. The heatmap showed flower density, floral size and floral shape of each species. Different color bar represent different families. The phylogenetic tree was constructed using *R* package ‘V.PhyloMaker’ and the heatmap of floral size and flower density was created in Evolview v3 (a webserver for visualization, annotation, and management of phylogenetic trees, https://www.evolgenius.info/)

Supplementary Figure 4 The correlation matrix between floral traits and network metrics. Size of square represents the value of correlation coefficient. *P* value (> 0.05) was shown in the square.

Supplementary Figure 5 Differences in five floral traits (floral size, flower density, floral shape, floral symmetry and floral color) among four different modules in the plant-pollinator network by a Tukey *post hoc* test using the *R* package ‘multcomp’ (Hothorn et al., 2014). **P* < 0.05, ** *P* < 0.01, *** *P* < 0.001.

Supplementary Figure 6 Differences in five floral traits (floral size, flower density, floral shape, floral symmetry and floral color) among peripheral species, connect species and network hubs in the plant-pollinator network, which were performed a Tukey *post hoc* test by the package ‘multcomp’ in *R*. * *P* < 0.05, ***P* < 0.01, ****P* < 0.001.

## References

[B1] Aizen M. A. Gleiser G. Sabatino M. Gilarranz L. J. Bascompte J. Verdú M. (2016). The phylogenetic structure of plant-pollinator networks increases with habitat size and isolation. Ecol. Lett. 19, 29–36. doi: 10.1111/ele.12539 26493295

[B2] Albor C. Ashman T. L. Stanley A. Martel C. Arceo-Gómez G. (2022). Flower colour and flowering phenology mediate plant-pollinator interaction assembly in a diverse co-flowering community. Funct. Ecol. 36, 2456–2468. doi: 10.1111/1365-2435.14142

[B3] Almeida-Neto M. Guimaraes P. Guimaraes P.R. Loyola R.D. Ulrich W. (2008). A consistent metric for nestedness analysis in ecological systems: reconciling concept and measurement. Oikos. 117, 1227–1239. doi: 10.1111/j.0030-1299.2008.16644.x

[B4] Bartomeus I. Ascher J. S. Gibbs J. Danforth B. N. Wagner D. L. Hedtke S. M.. (2013). Historical changes in northeastern US bee pollinators related to shared ecological traits. Proc. Natl. Acad. Sci. U. S. A. 110, 4656–4660. doi: 10.1073/pnas.1218503110 23487768 PMC3606985

[B5] Barton K. (2018). “MuMIn: Multi-model inference,” in R package version 1.40.4. Available at: https://CRAN.R-project.org/package.

[B6] Bascompte J. Jordano P. (2007). Plant-animal mutualistic networks: the architecture of biodiversity. Annu. Rev. Ecol. Evol. S 101, 567–593. doi: 10.1146/annurev.ecolsys.38.091206.095818

[B7] Bascompte J. Jordano P. Melian C. J. Olesen J. M. (2003). The nested assembly of plant-animal mutualistic networks. Proc. Natl. Acad. Sci. U. S. A. 100, 9383–9387. doi: 10.1073/pnas.1633576100 12881488 PMC170927

[B8] Blüthgen N. Menzel F. Blüthgen N. (2006). Measuring specialization in species interaction networks. BMC Ecol. 6, 9. doi: 10.1186/1472-6785-6-96 16907983 PMC1570337

[B9] Blomberg S. P. Garland T. Ives A. R. (2003). Testing for phylogenetic signal in comparative data-behavioral traits are more labile. Evolution 57, 717–745. doi: 10.1111/j.0014-3820.2003.tb00285.x 12778543

[B10] Campbell D. R. Bischoff M. Lord J. M. Robertson A. W. (2012). Where have all the blue flowers gone: pollinator responses and selection on flower colour in new Zealand *Wahlenbergia albomarginata* . J. Evol. Biol. 25, 352–364. doi: 10.1111/j.1420-9101.2011.02430.x 22151952

[B11] Carstensen D. W. Sabatino M. Morellato L. P. (2016). Modularity, pollination systems, and interaction turnover in plant-pollinator networks across space. Ecology 97, 1298–1306. doi: 10.1890/15-0830.1 27349105

[B12] Carstensen D. W. Sabatino M. Trøjelsgaard K. Morellato L. P. C. (2014). Beta diversity of plant-pollinator networks and the spatial turnover of pairwise interactions. PloS One 9, e112903. doi: 10.1371/journal.pone.0112903 25384058 PMC4226610

[B13] Cartar R. V. (2009). Resource-tracking by bumble bees: What explains local responses to density of bergamot (*Monarda fistulosa*) flowers? Ecoscience 16, 470–475. doi: 10.2980/16-4-3209

[B14] Carvalheiro L. G. Biesmeijer J. C. Benadi G. Fründ J. Stang M. Bartomeus I.. (2014). The potential for indirect effects between co-flowering plants *via* shared pollinators depends on resource abundance, accessibility and relatedness. Ecol. Lett. 17, 1389–`1399. doi: 10.1111/ele.12342 25167890

[B15] Chamberlain S. A. Cartar R. V. Worley A. C. Semmler S. J. Gielens G. Elwell S.. (2014). Traits and phylogenetic history contribute to network structure across Canadian plant-pollinator communities. Oecologia 176, 545–556. doi: 10.1007/s00442-014-3035-2 25142045

[B16] Chesshire P. R. McCabe L. M. Cobb N. S. (2021). Variation in Plant–Pollinator Network Structure along the Elevational Gradient of the San Francisco Peaks, Arizona. Insects 12, 1060. doi: 10.3390/insects12121060 34940148 PMC8704280

[B17] Chittka L. Raine N. E. (2006). Recognition of flowers by pollinators. Curr. Opin. Plant Biol. 9 (4), 428–435. doi: 10.1016/j.pbi.2006.05.002 16713328

[B18] Cirtwill A. R. Dalla Riva G. V. Baker N. J. Ohlsson M. Norström I. Wohlfarth I. M.. (2020). Related plants tend to share pollinators and herbivores, but strength of phylogenetic signal varies among plant families. New Phyto. 226 (3), 909–920. doi: 10.1111/nph.16420 31917859

[B19] Dellinger A. S. Artuso S. Pamperl S. Michelangeli F. A. Penneys D. S. Fernandez-Fernandez D. M. (2019). Modularity increases rate of floral evolution and adaptive success for functionally specialized pollination systems. Commun. Biol. 2, 453. doi: 10.1038/s42003-019-0697-7 31872071 PMC6895197

[B20] Dellinger A. S. (2020). Pollination syndromes in the 21st century: where do we stand and where may we go? New Phyto 228, 1193–1213. doi: 10.1111/nph.16793 33460152

[B21] Doré M. Fontaine C. Thébault E. (2021). Relative effects of anthropogenic pressures, climate, and sampling design on the structure of pollination networks at the global scale. Glob. Change Biol. 27, 1266–1280. doi: 10.1111/gcb.15474 33274540

[B22] Dormann C. F. Fründ J. Blüthgen N. Gruber B. (2009). Indices, graphs and null models: Analyzing bipartite ecological networks. Open Ecol. J. 2, 7–24. doi: 10.2174/1874213000902010007

[B23] Dormann C. F. Strauss R. (2014). A method for detecting modules in quantitative bipartite networks. Methods Ecol. Evol. 5, 90–98. doi: 10.1111/2041-210X.12139

[B24] Faegri K. Van der Pijl L. (1979). The principles of pollination ecology (Oxford: Pergamon Press).

[B25] Fontaine C. Dajoz I. Meriguet J. Loreau M. (2006). Functional diversity of plant–pollinator interaction webs enhances the persistence of plant communities. PloS Biol. 4, 129–135. doi: 10.1371/journal.pbio.0040001 PMC1310649 16332160

[B26] Fortuna M. A. Stouffer D. B. Olesen J. M. Jordano P. Mouillot D. Krasnov B. R.. (2010). Nestedness versus modularity in ecological networks: two sides of the same coin? J. Anim. Ecol., 79, 811–817. doi: 10.1111/j.1365-2656.2010.01688.x 20374411

[B27] Gaiarsa M. P. Bascompte J. (2022). Hidden effects of habitat restoration on the persistence of pollination networks. Ecol. Lett. 5, 2132–2141. doi: 10.1111/ele.14081 PMC9804604 36006740

[B28] Gallai N. Salles J. M. Settele J. Vaissière B. E. (2009). Economic valuation of the vulnerability of world agriculture confronted with pollinator decline. Ecol. Econ. 68, 810–821. doi: 10.1016/j.ecolecon.2008.06.014

[B29] Garland T. Dickerman A. W. Janis C. M. Jones J. A. (1993). Phylogenetic analysis of covariance by computer simulation. Syst. Biol. 42, 265–292. doi: 10.1093/sysbio/42.3.265

[B30] Geslin B. Gauzens B. Thebault E. Dajoz I. (2013). Plant pollinator networks along a gradient of urbanisation. PloS One 8, e63421. doi: 10.1371/journal.pone.0063421 23717421 PMC3661593

[B31] Gibson M. R. Richardson D. M. Pauw A. Gibson D. (2012). Can floral traits predict an invasive plant's impact on native plant-pollinator communities? J. Ecol. 100, 1216–1223. doi: 10.1111/j.1365-2745.2012.02004.x

[B32] Gómez J. M. Perfectti F. (2010). Evolution of complex traits: the case of *Erysimum* corolla shape. Int. J. Plant Sci. 171, 987–998. doi: 10.1086/656475

[B33] González-Varo J. P. Biesmeijer J. C. Bommarco R. Potts S. G. Schweiger O. Smith H. G.. (2013). Combined effects of global change pressures on animal-mediated pollination. Trends Ecol. Evol. 28 (9), 524–530. doi: 10.1016/j.tree.2013.05.008 23746938

[B34] Gu Z. Eils R. Schlesner M. (2016). Complex heatmaps reveal patterns and correlations in multidimensional genomic data. Bioinformatics, 32, 2847–2849. doi: 10.1093/bioinformatics/btw313 27207943

[B35] Guimarães P. R. Pires M. M. Jordano P. Bascompte J. Thompson J. N. (2017). Indirect effects drive coevolution in mutualistic networks. Nature 550, 511–514. doi: 10.1038/nature24273 29045396

[B36] Guimeraà R. Mossa S. Turtschi A. Amaral L. A. N. (2005). The world- wide air transportation network: Anomalous centrality, community structure, and cities' global roles. Proc. Natl. Acad. Sci. U.S.A. 102, 7794–7799. doi: 10.1073/pnas.0407994102 15911778 PMC1142352

[B37] Harmon L. J. Weir J. T. Brock C. D. Glor R. E. Challenger W. (2008). GEIGER: investigating evolutionary radiations. Bioinformatics 24, 129–131. doi: 10.1093/bioinformatics/btm538 18006550

[B38] He N. Liu C. Piao S. Sack L. Xu L. Luo Y.. (2019). Ecosystem traits linking functional traits to macroecology. Trends Ecol. Evol. 34 (3), 200–210. doi: 10.1016/j.tree.2018.11.004 30527959

[B39] Junker R. R. Blüthgen N. Brehm T. Binkenstein J. Paulus J. Martin Schaefer H.. (2013). Specialization on traits as basis for the niche-breadth of flower visitors and as structuring mechanism of ecological networks. Funct. Ecol. 27, 329–341. doi: 10.1111/1365-2435.12005

[B40] Kantsa A. Raguso R. A. Dyer A. G. Olesen J. M. Tscheulin T. Petanidou T. (2018). Disentangling the role of floral sensory stimuli in pollination networks. Nat. Commun. 9, 1041. doi: 10.1038/s41467-018-03448-w 29531220 PMC5847531

[B41] Kembel S. W. Cowan P. D. Helmus M. R. Cornwell W. K. Morlon H. Ackerly D. D.. (2010). Picante: R tools for integrating phylogenies and ecology. Bioinformatics 26, 1463–1464. doi: 10.1093/bioinformatics/btq166 20395285

[B42] Koski M. H. Meindl G. A. Arceo-Gómez G. Wolowski M. LeCroy K. A. Ashman T. L. (2015). Plant-flower visitor networks in a serpentine metacommunity: assessing traits associated with keystone plant species. Arthr. Plant Interact. 9, 9–21. doi: 10.1007/s11829-014-9353-9

[B43] Kunin W. E. (1997). Population size and density effects in pollination: pollinator foraging and plant reproductive success in experimental arrays of *Brassica kaber* . J. Ecol. 85, 225–234. doi: 10.2307/2960653

[B44] Lara‐Romero C. Seguí J. Pérez‐Delgado A. Nogales M. Traveset A. (2019). Beta diversity and specialization in plant–pollinator networks along an elevational gradient. J. Biogeogr. 46, 1598–1610. doi: 10.1111/jbi.13615

[B45] Lázaro A. Gómez-Martínez C. Alomar D. González-Estévez M. A. Traveset A. Rafferty N. (2020). Linking species-level network metrics to flower traits and plant fitness. J. Ecol. 108, 1287–1298. doi: 10.1111/1365-2745.13334

[B46] Lefcheck J. S. (2016). piecewiseSEM: Piecewise structural equation modelling in r for ecology, evolution, and systematics. Methods Ecol. Evol. 7, 573–579. doi: 10.1111/2041-210x.12512

[B47] Li W. Y. Ai X. R. Yao L. Zhu J. (2021). Biodiversity evaluation of three national nature reserve in southwest hubei. Hubei For. Sci. Tech. 50, 18–22.

[B48] Makino T. T. Ohashi K. Sakai S. (2007). How do floral display size and the density of surrounding flowers influence the likelihood of bumble bee revisitation to a plant? Funct. Ecol. 21, 87–95. doi: 10.1111/j.1365-2435.2006.01211.x

[B49] Man J. S. Peng Z. L. Fang Y. P. Liu S. X. (2008). Study on the wild plant resource in qizimeishan national nature preserve and its exploitation. J. Anhui Agr. Sci. 12, 5119–5120. doi: 10.13989/j.cnki.0517-6611.2008.12.011

[B50] Martins E. P. Hansen T. F. (1997). Phylogenies and the comparative method: a general approach to incorporating phylogenetic information into the analysis of interspecific data. Am. Nat. 149, 646–667. doi: 10.1086/286013

[B51] Moeller D. A. (2004). Facilitative interactions among plants *via* shared pollinators. Ecology 85, 3289–3301. doi: 10.1890/03-0810

[B52] Olesen J. M. Bascompte J. Dupont Y. L. Jordano P. (2007). The modularity of pollination networks. Proc. Natl. Acad. Sci. U. S. A. 104, 19891–19896. doi: 10.1073/pnas.0706375104 18056808 PMC2148393

[B53] Ollerton J. Winfree R. Tarrant S. (2011). How many flowering plants are pollinated by animals? Oikos 120, 321–326. doi: 10.1111/j.1600-0706.2010.18644.x

[B54] Opsahl T. Agneessens F. Skvoretz J. (2010). Node centrality in weighted networks: Generalizing degree and shortest paths. Soc Network. 32, 245–251. doi: 10.1016/j.socnet.2010.03.006

[B55] Ouvrard P. Transon J. Jacquemart A.-L. (2018). Flower-strip agri-environment schemes provide diverse and valuable summer flower resources for pollinating insects. Biodivers. Conserv. 27, 2193–2216. doi: 10.1007/s10531-018-1531-0

[B56] Pagel M. (1999). Inferring the historical patterns of biological evolution. Nature 401, 877–884. doi: 10.1038/44766 10553904

[B57] Pardo-De la Hoz C. J. Medeiros I. D. Gibert J. P. Chagnon P. L. Magain N. Miadlikowska J.. (2022). Phylogenetic structure of specialization: A new approach that integrates partner availability and phylogenetic diversity to quantify biotic specialization in ecological networks. Ecol. Evol. 12, e8649. doi: 10.1002/ece3.8649 35261742 PMC8888259

[B58] Petanidou T. Kallimanis A. S. Tzanopoulos J. Sgardelis S. P. Pantis J. D. (2008). Long-term observation of a pollination network: fluctuation in species and interactions, relative invariance of network structure and implications for estimates of specialization. Ecol. Lett. 11, 564–575. doi: 10.1111/j.1461-0248.2008.01170.x 18363716

[B59] Pocock M. J. O. Johnson O. Wasiuk D. (2011). Succinctly assessing the topological importance of species in flower–pollinator networks. Ecol. Complex. 8, 265–272. doi: 10.1016/j.ecocom.2011.06.003

[B60] Qian H. Jin Y. (2016). An updated megaphylogeny of plants, a tool for generating plant phylogenies and an analysis of phylogenetic community structure. J. Plant Ecol. 9, 233–239. doi: 10.1093/jpe/rtv047

[B61] R Core Team (2022). R: A language and environment for statistical computing (Vienna, Austria: R Foundation for Statistical Computing). Available at: https://www.R-project.org/.

[B62] Reginato M. Michelangeli F. A. (2016). Diversity and constraints in the floral morphological evolution of *Leandra* s.str. (Melastomataceae). Ann. Bot. 118, 445–458. doi: 10.1093/aob/mcw116 27401539 PMC4998978

[B63] Reverté S. Retana J. Gómez J. M. Bosch J. (2016). Pollinators show flower colour preferences but flowers with similar colours do not attract similar pollinators. Ann. Bot. 118, 249–257. doi: 10.1093/aob/mcw103 27325897 PMC4970366

[B64] Rezende E. L. Jordano P. Bascompte J. (2007a). Effects of phenotypic complementarity and phylogeny on the nested structure of mutualistic networks. Oikos 116, 1919–1929. doi: 10.1111/j.0030-1299.2007.16029.x

[B65] Rezende E. L. Lavabre J. E. Guimarães P. R. Jordano P. Bascompte J. (2007b). Non-random coextinctions in phylogenetically structured mutualistic networks. Nature 448, 925–928. doi: 10.1038/nature05956 17713534

[B66] Saavedra S. Stouffer D. B. Uzzi B. Bascompte J. (2011). Strong contributors to network persistence are the most vulnerable to extinction. Nature 478, 233–235. doi: 10.1038/nature10433 21918515

[B67] Sazima C. Guimarães P. R. dos Reis S. F. Sazima I. (2010). What makes a species central in a cleaning mutualism network? Oikos 119, 1319–1325. doi: 10.1111/j.1600-0706.2009.18222.x

[B68] Segar S. T. Fayle T. M. Srivastava D. S. Lewinsohn T. M. Lewis O. T. Novotny V.. (2020). The role of evolution in shaping ecological networks. Trends. Ecol. Evol. 35, 454–466. doi: 10.1016/j.tree.2020.01.004 32294426

[B69] Shipley B. (2009). Confirmatory path analysis in a generalized multilevel context. Ecology. 90, 363—368. doi: 10.1890/08-1034.1 19323220

[B70] Shipley B. (2013). The AIC model selection method applied to path analytic models compared using a d-separation test. Ecology. 94, 560–564. doi: 10.1890/12-0976.1 23687881

[B71] Shrestha M. Dyer A. G. Bhattarai P. Burd M. (2014). Flower colour and phylogeny along an altitudinal gradient in the Himalayas of Nepal. J. Ecol. 102, 126–135. doi: 10.1111/1365-2745.12185

[B72] Stang M. Klinkhamer P. G. van der Meijden E. (2006). Size constraints and flower abundance determine the number of interactions in a plant-flower visitor web. Oikos 112, 111–121. doi: 10.1111/j.0030-1299.2006.14199.x

[B73] Stang M. Klinkhamer P. G. L. van der Meijden E. (2007). Asymmetric specialization and extinction risk in plant–flower visitor webs: A matter of morphology or abundance? Oecologia 151, 442–453. doi: 10.1007/s00442-006-0585-y 17080257

[B74] Stang M. Klinkhamer P. G. L. Waser N. M. Stang I. van der Meijden E. (2009). Size-specific interaction patterns and size matching in a plant–pollinator interaction web. Ann. Bot. 103, 1459–1469. doi: 10.1093/aob/mcp027 19228701 PMC2701768

[B75] Stebbins G. L. (1970). Adaptive radiation of reproductive characteristics in angiosperms. I: pollination mechanisms. Annu. Rev. Ecol. Syst., 1, 307–326. doi: 10.1146/annurev.es.01.110170.001515

[B76] Suárez-Mariño A. Arceo-Gómez G. Albor C. Parra-Tabla V. (2022). Flowering overlap and floral trait similarity help explain the structure of pollination networks. J. Ecol. 110, 1790–1801. doi: 10.1111/1365-2745.13905

[B77] Tinoco B. A. Graham C. H. Aguilar J. M. Schleuning M. (2017). Effects of hummingbird morphology on specialization in pollination networks vary with resource availability. Oikos 126, 52–60. doi: 10.1111/oik.02998

[B78] Trøjelsgaard K. Heleno R. Traveset A. (2019). Native and alien flower visitors differ in partner fidelity and network integration. Ecol. Lett. 22, 1264–1273. doi: 10.1111/ele.13287 31148310

[B79] Villalobos S. Sevenello-Montagner J. M. Vamosi J. C. (2019). Specialization in plant-pollinator networks: insights from local-scale interactions in glenbow ranch provincial park in Alberta, Canada. BMC Ecol. 19, 34. doi: 10.1186/s12898-019-0250-z 31492127 PMC6731600

[B80] Violle C. Navas M. L. Vile D. Kazakou E. Fortunel C. Hummel I. Garnier E. (2007). Let the concept of trait be functional! Oikos 116, 882–892. doi: 10.1111/j.0030-1299.2007.15559.x

[B81] Watts S. Dormann C. F. Martin Gonzalez A. M. Ollerton J. (2016). The influence of floral traits on specialization and modularity of plant-pollinator networks in a biodiversity hotspot in the Peruvian Andes. Ann. Bot. 118, 415–429. doi: 10.1093/aob/mcw114 27562649 PMC4998976

[B82] Wei N. Kaczorowski R. L. Arceo-Gomez G. O'Neill E. M. Hayes R. A. Ashman T. L. (2021). Pollinators contribute to the maintenance of flowering plant diversity. Nature 597, 688–692. doi: 10.1038/s41586-021-03890-9 34497416

[B83] Wei T. Simko V. (2017) Corrplot: Visualization of a correlation matrix. r package version 0.84. Available at: https://CRAN.R-project.org/package=corrplot.

[B84] Wignall A. E. Heiling A. M. Cheng K. Herberstein M. E. (2006). Flower symmetry preferences in honeybees and their crab spider predators. Ethology 112, 510–518. doi: 10.1111/J.1439-0310.2006.01199.X

[B85] Willmer P. (2011). Pollination and floral ecology (Princeton, NJ: Princeton University Press).

[B86] Zhao Y. H. Lázaro A. Ren Z. X. Zhou W. Li H. D. Tao Z. B.. (2019). The topological differences between visitation and pollen transport networks: a comparison in species rich communities of the himalaya-hengduan mountains. Oikos 128, 551–562. doi: 10.1111/oik.05262

