# Supplementary figures and images for: Different influences of phylogenetically conserved and independent floral traits on plant functional specialization and pollination network structure
Source: Front Plant Sci. 2023 Jan 24;14:1084995. doi: 10.3389/fpls.2023.1084995 (PMC9902514; doi:10.3389/fpls.2023.1084995)

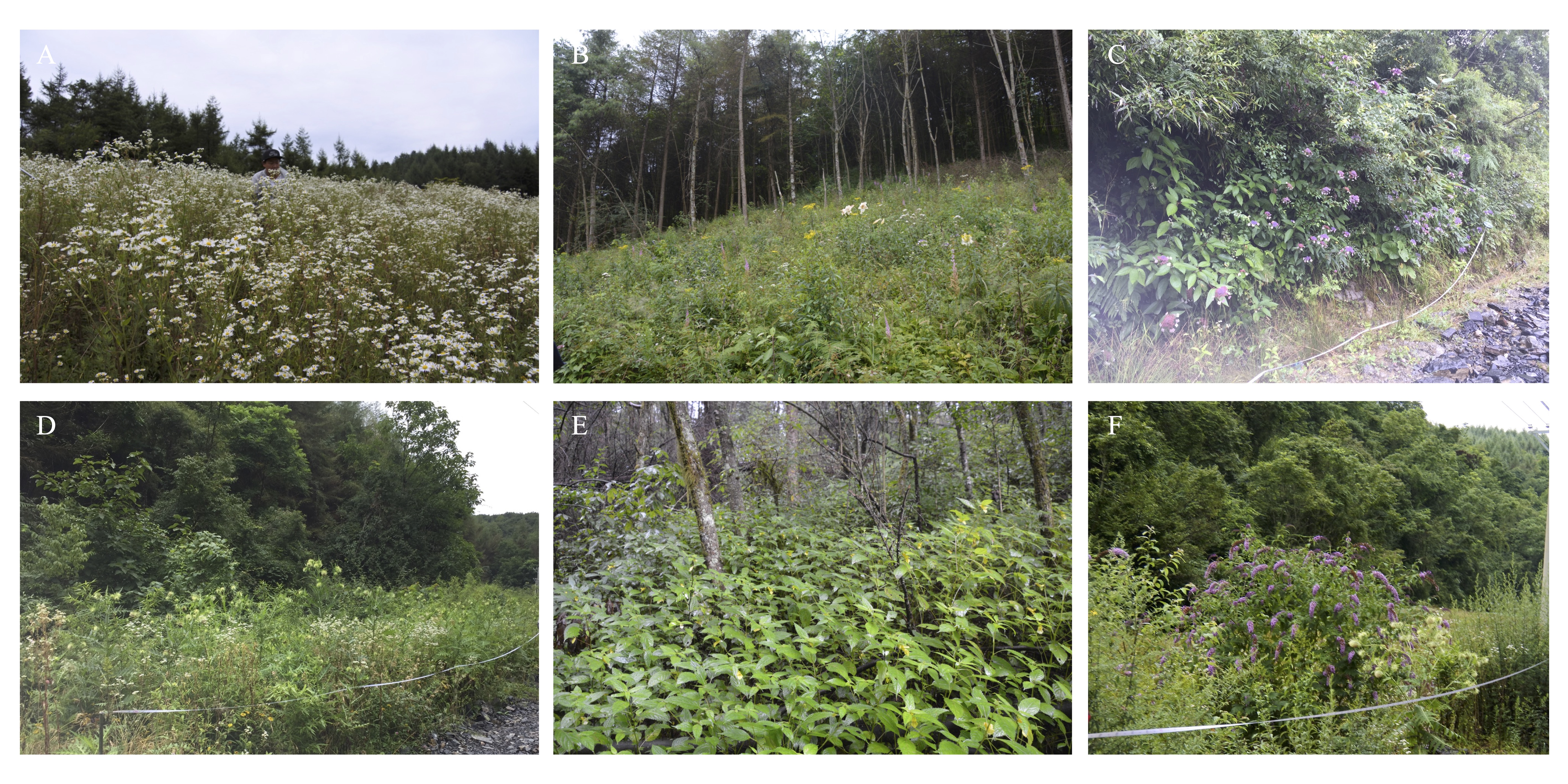

Supplement: Supplementary Figure 1 — Study sites, grassland plots (A, B) and roadside plots (C, D, E, F) in Hubei Qizimeishan National Nature Reserve, China. [file Image_1.jpeg]

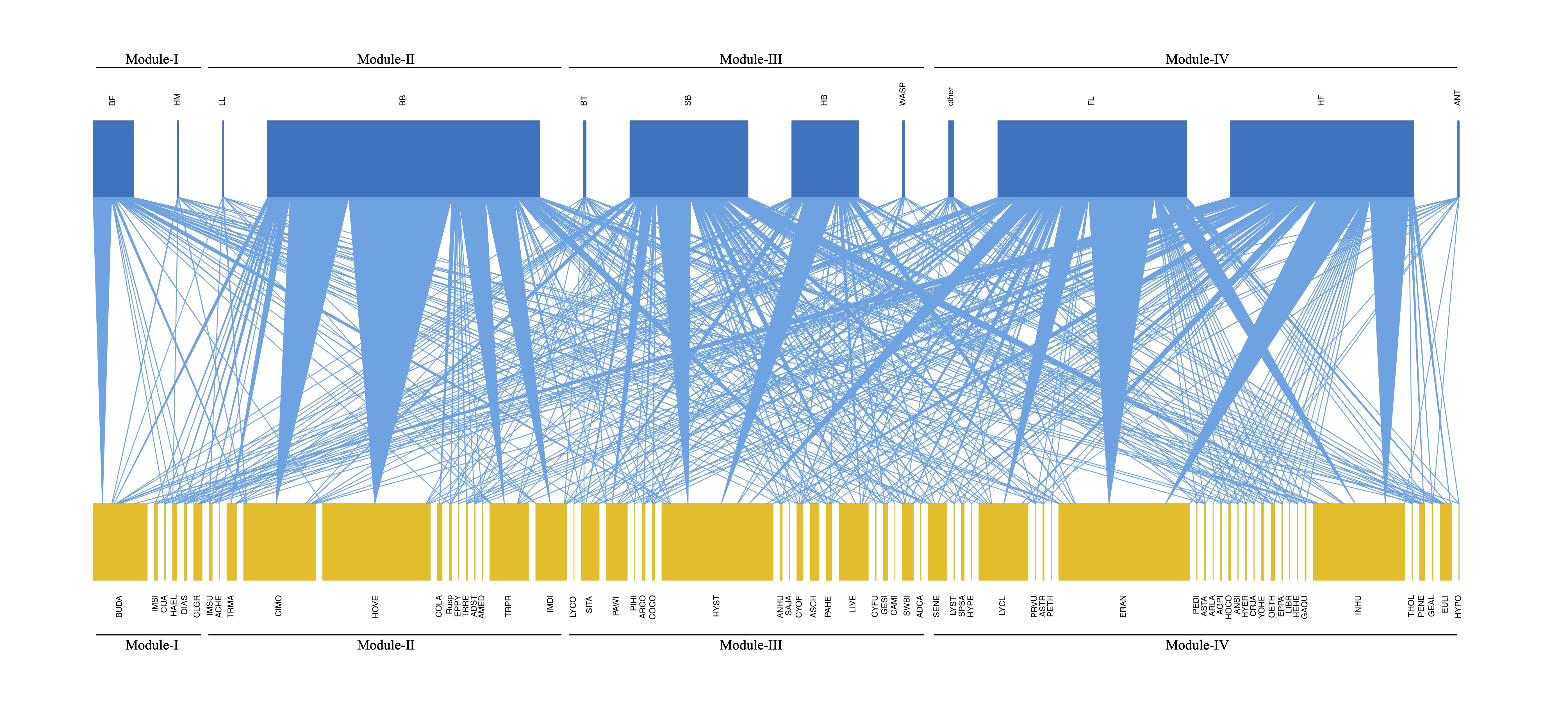

Supplement: Supplementary Figure 2 — The plant-pollinator network in our community with pollinator at the top and plants at the bottom. The widths of the lines connecting plants with their pollinators represents the number of flowers visited by each pollinator. Plant species were shown as plant code (See Supplementary Table 1 for each species’ code). Pollinator and plant species were sequenced according to the module structure. [file Image_2.jpeg]

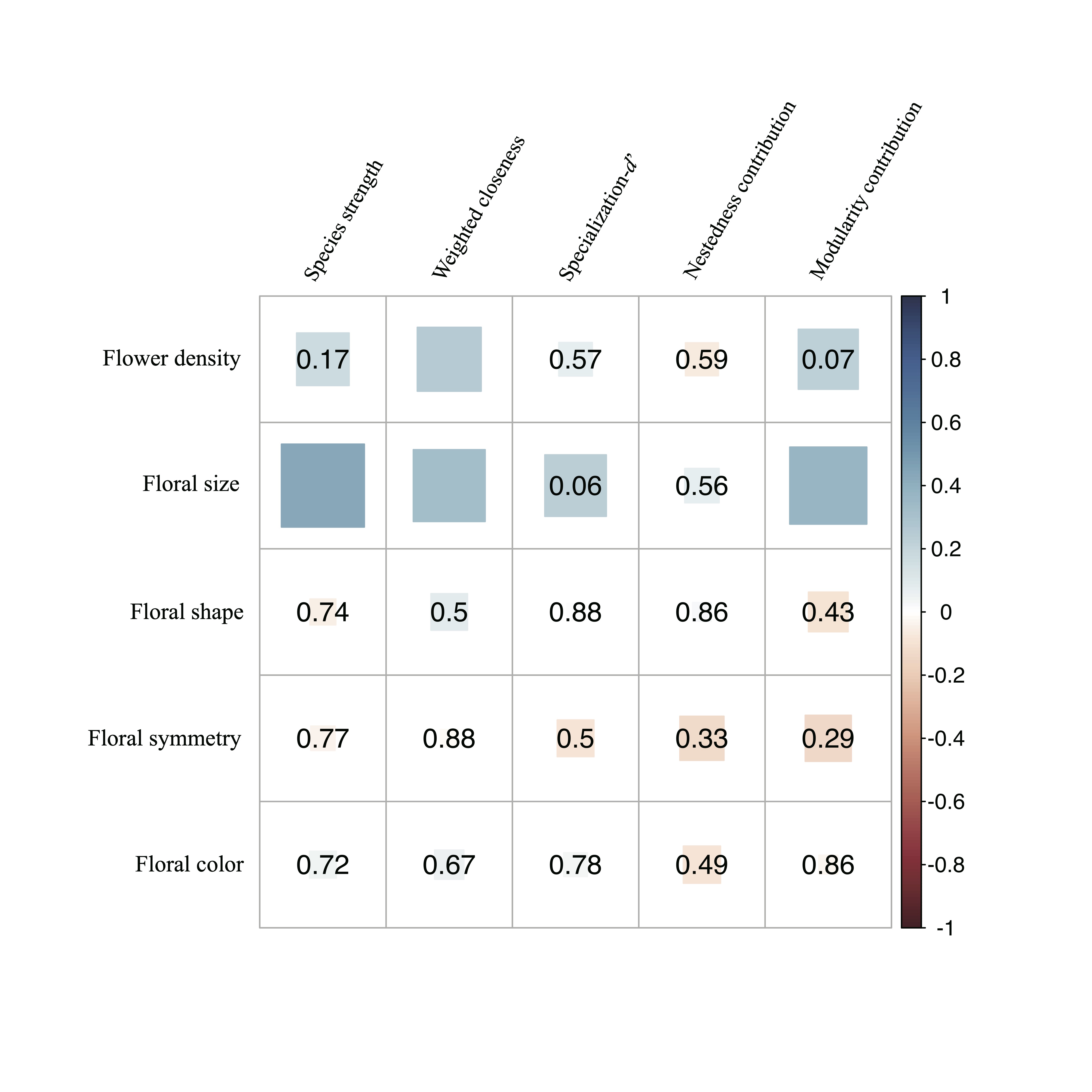

Supplement: Supplementary Figure 4 — The correlation matrix between floral traits and network metrics. Size of square represents the value of correlation coefficient. P value (> 0.05) was shown in the square. [file Image_4.jpeg]

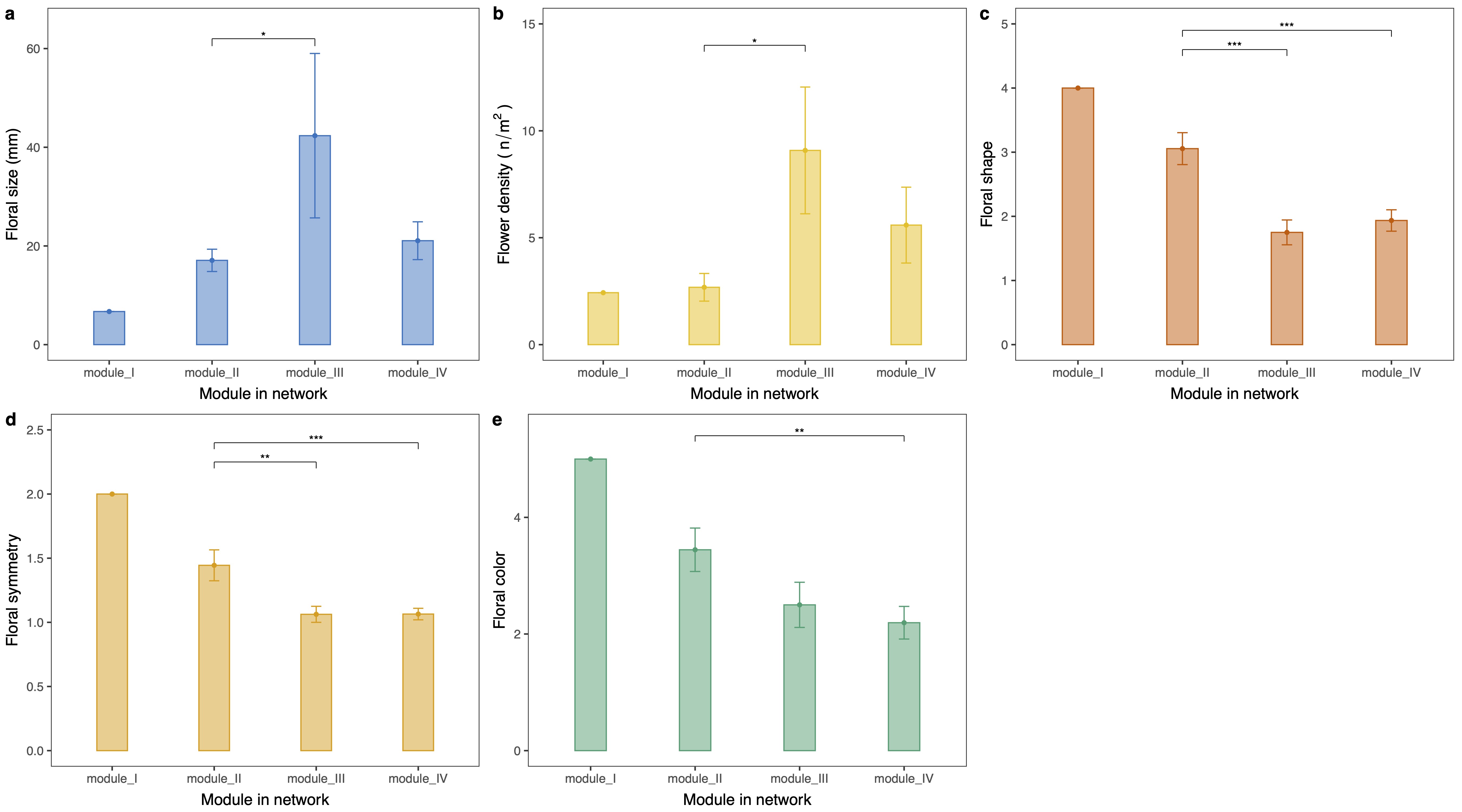

Supplement: Supplementary Figure 5 — Differences in five floral traits (floral size, flower density, floral shape, floral symmetry and floral color) among four different modules in the plant-pollinator network by a Tukey post hoc test using the R package ‘multcomp’ (Hothorn et al., 2014). *P < 0.05, ** P < 0.01, *** P < 0.001. [file Image_5.jpeg]

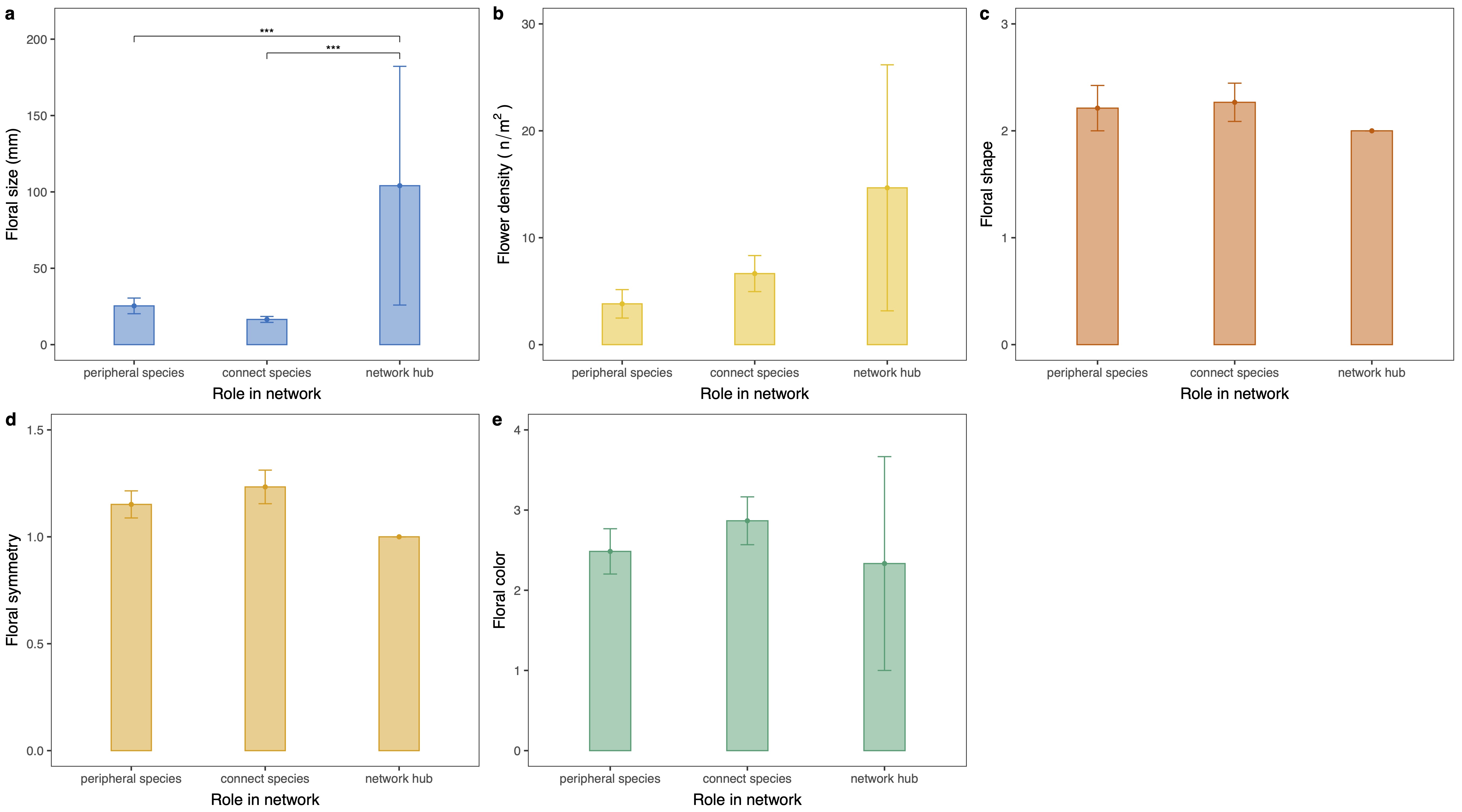

Supplement: Supplementary Figure 6 — Differences in five floral traits (floral size, flower density, floral shape, floral symmetry and floral color) among peripheral species, connect species and network hubs in the plant-pollinator network, which were performed a Tukey post hoc test by the package ‘multcomp’ in R. * P < 0.05, **P < 0.01, ***P < 0.001. [file Image_6.jpeg]
