# Supplementary material for: Different influences of phylogenetically conserved and independent floral traits on plant functional specialization and pollination network structure
Source: Front Plant Sci. 2023 Jan 24;14:1084995. doi: 10.3389/fpls.2023.1084995 (PMC9902514; doi:10.3389/fpls.2023.1084995)
Supplement: Supplementary file 9 [file Table_3.docx]

**Supplementary Table 3** Description of floral visitor functional groups observed visiting plants in our study sites

| Floral visitor functional groups | Pollination code | description |
| --- | --- | --- |
| Bumblebees | BB | Bees within the genus *Bombus*, social, efficient forager of pollen and nectar, efficient recruiter, pollen carried on hind legs |
| Large solitary bees | LL | Native bees within the large bodies, solitary, forage on pollen and nectar, pollen carried on hind legs |
| Honeybees | HB | Specific only *Apis cerana* according to the field observation, mostly domesticated by villagers near their house social, efficient forager of pollen and nectar, efficient recruiter, pollen carried on hind legs |
| Small bees | SB | Bees with small body size, ectothermic, fast flyer, solitary or social, pollen carried on hind legs or the underside of abdomen, including members of Halictidae, Andrenidae, Apidae, Megachilidae |
| Wasps | WASP | Mostly carnivorous, some groups visit for nectar, pollen not carried on specific location on body, including members of Vespidae |
| Ants | Ant | Walking insects, pollen not carried on specific location on body, including members of Formicidae |
| Hoverflies | HF | Fast flying flies in family Syrphidae with variable feeding time and long foraging season, foraging on pollen and nectar, pollen not carried on specific location on body |
| Other flies | FL | Other flies include members of Tachinidae, Muscidae and Calliphoridae, different from syrphid flies, small, inefficient, pollen not carried on specific location on body |
| Butterflies | BF | Lepidoptera species except hawkmoths, nectar foragers, long foraging times, fly long distances, narrow thermal requirements, forage on low concentrated nectar, pollen not carried on specific location on body |
| Hawk moths | HM | Species in Sphingidae (Lepidoptera), rapid and sustained flying, hover in midair while feeding on nectar from flowers |
| Beetles | BT | mostly pollen eaters, inefficient, short flying range, pollen not carried in specific location on body, including Scarabaeidae, Cantharidae, Cleridae and Buprestidae (Coleoptera), |
| Other insects | Others | Staying insects includes members of Pentatomoidea and other tiny bugs, pollen not carried on specific location on body, mostly with limited pollination efficiency |
